# A case of primary nonleukemic myeloid sarcoma of the spleen, successfully treated by surgery and hematopoietic stem cell transplantation

**DOI:** 10.1186/s40792-021-01257-w

**Published:** 2021-08-11

**Authors:** Asuka Ono, Yuki Kitano, Katsunori Imai, Takashi Matsumoto, Shinya Endo, Kenji Tokunaga, Hiromitsu Hayashi, Yo-Ichi Yamashita, Masao Matsuoka, Hideo Baba

**Affiliations:** 1 Department of Gastroenterological Surgery, Graduate School of Medical Sciences, Kumamoto University, 1-1-1 Honjo, Chuo-ku, Kumamoto, 860-8556 Japan; 2 Departments of Hematology, Rheumatology, and Infectious Disease, Graduate School of Medical Sciences, Kumamoto University, Kumamoto, Japan

**Keywords:** Myeloid sarcoma, Spleen, Surgery, Nonleukemic

## Abstract

**Background:**

Myeloid sarcoma (MS) is a rare disease, mostly found in conjunction with acute myelogenous leukemia or other diseases, and primary nonleukemic MS of the spleen is particularly rare.

**Case presentation:**

We report a 57-year-old male who presented with a spleen mass that was found incidentally, and was enlarged. As a result of various examinations, he was diagnosed with primary MS of the spleen with suspected involvement of the transverse colon, left kidney, pancreatic tail, and left diaphragm. He underwent a total splenectomy, partial pancreatectomy, partial colectomy, left nephrectomy, and left diaphragm partial resection. Histological examination revealed splenic primary MS. Bone marrow biopsy and immunophenotypic flow cytometry revealed no evidence of myeloid leukemia. He underwent umbilical cord blood transplantation, and he is currently living without a sign of recurrence at 10 months after surgery.

**Conclusions:**

We experienced a very rare case of primary spleen MS that was discovered without a hematologic malignancy. Two cases of surgically resected primary splenic MS have been reported, including the present case.

## Background

Myeloid sarcoma (MS) is defined as an extramedullary tumor consisting of myeloid blasts in one or more of the myeloid lineages, and it disrupts the normal architecture of the tissue. MS often occurs concomitantly with acute myeloid leukemia (AML) and rarely without bone marrow involvement. Isolated MS is a rare entity with an incidence of two cases per million adults, and the incidence of MS is 2.5–9% of patients with AML and less than 1% without bone marrow involvement [1, 2]. MS may occur at any site of the body, although frequent sites are the lymph nodes, skin, soft tissues, testes, bone, peritoneum, and the gastrointestinal tract [2]. In AML, MS may be the first symptom, and may precede clinical disease by months or years [3]. As far as we know to date, only one case has been reported as surgical resected primary spleen MS [4]. In this report, we describe a case of primary nonleukemic MS of the spleen that was successfully treated by surgery and hematopoietic stem cell transplantation.

## Case presentation

**Fig. 1:**
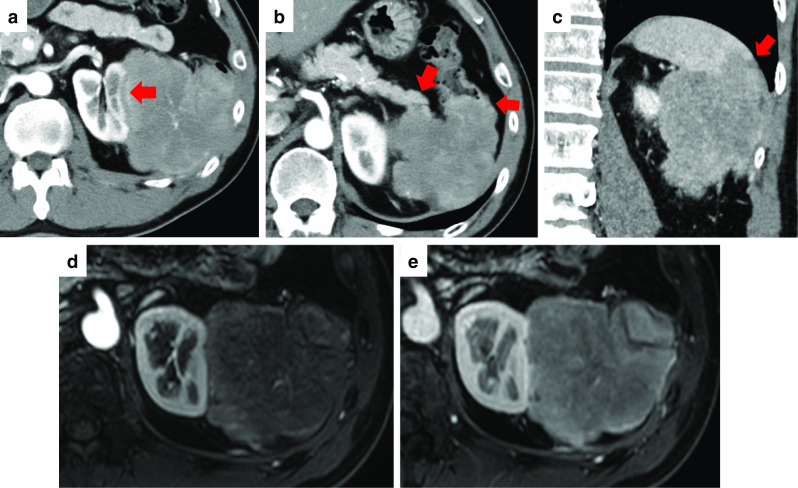
**a**–**c** Abdominal contrast-enhanced CT scan showed a huge mass in the spleen. The red arrows point out the involvement of surrounding organs: **a** left kidney, **b** pancreatic tail and descending colon, **c** left diaphragm. **d** Early phase of abdominal contrast-enhanced MRI. **e** Late phase of abdominal contrast-enhanced MRI

**Fig. 2:**
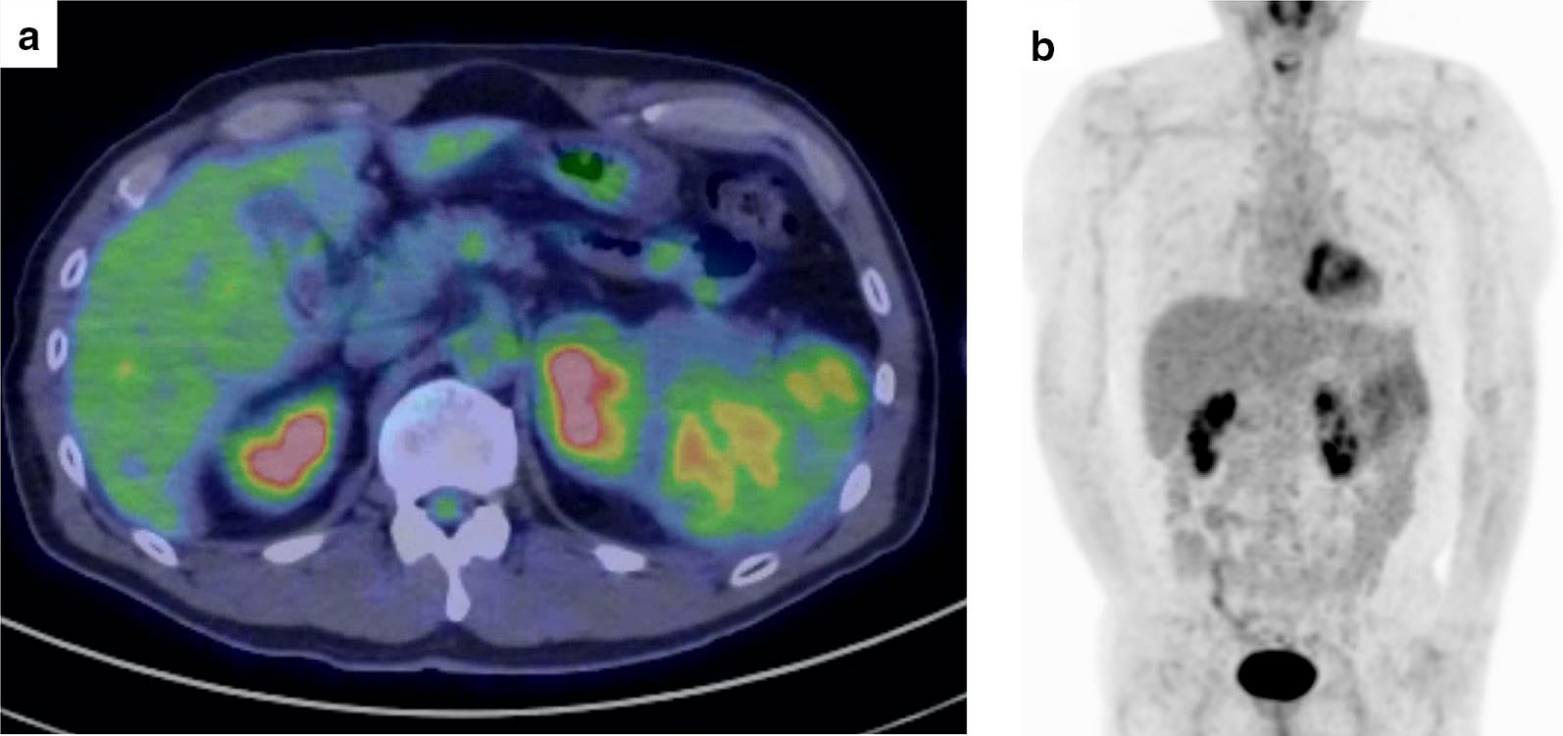
**a** PET–CT showed an accumulation of standardized uptake value (SUV) max of 4.1. **b** PET–CT showed no obvious lymph node or distant metastasis

**Fig. 3:**
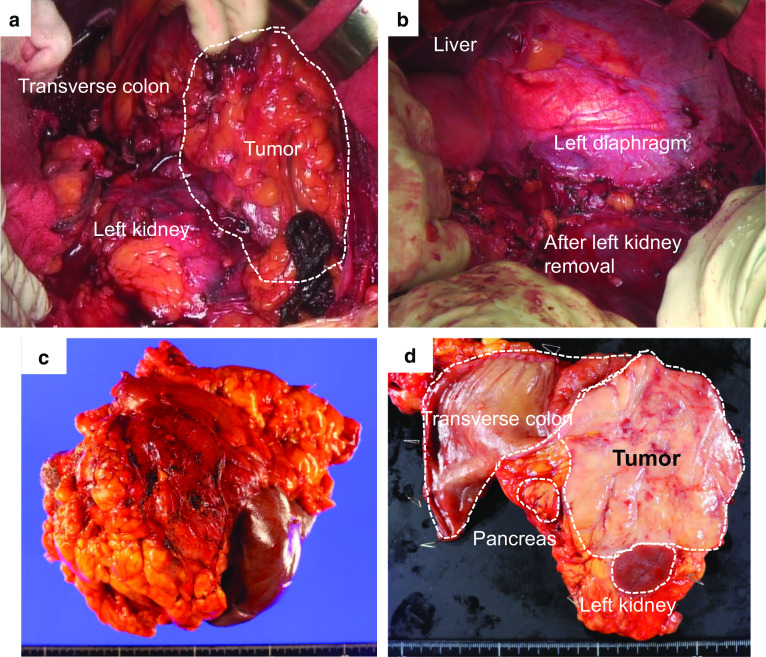
**a** Intraoperative finding before tumor removal. **b** Intraoperative finding after tumor removal. **c** A resected specimen. **d** A opened specimen

**Fig. 4:**
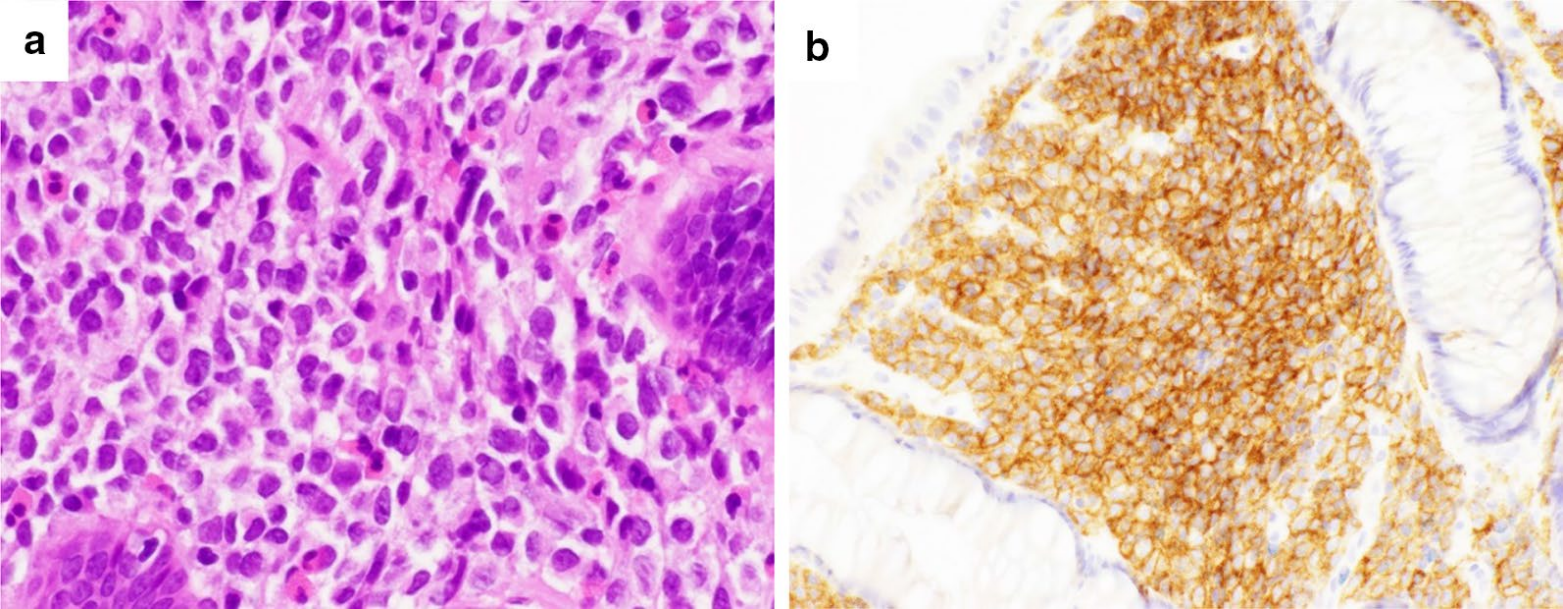
**a** Hematoxylin staining shows atypical cells with irregular karyotype and prominent nucleoli. **b** Immunohistochemical staining of CD33 is positive

The patient was a 57-year-old male. He did not have any symptoms, but he had been noted one year previously to have a spleen mass whose diameter was 41 mm. It was followed up because most spleen tumors were benign. The patient was admitted to hospital and underwent tooth extraction and cyst fenestration for a left odontogenic cyst. On the day after the surgery, he had a fever and complained of abdominal pain, and an abdominal contrast-enhanced computed tomography (CT) scan revealed acute cholecystitis with gallstones. At that time, an 85-mm mass was incidentally found in his spleen, and it had grown larger than before. Laboratory examination showed that his hemoglobin was 12.5 g/L, total leukocyte count was 5.2 × 10^9^/L, platelet count was 328 × 10^9^/L, and soluble interleukin-2 receptor was 322 U/mL. Abdominal contrast-enhanced CT and magnetic resonance imaging (MRI) showed a huge, irregularly shaped, and heterogeneously enhanced mass in the spleen, with findings of suspected involvement of the transverse colon, left kidney, pancreatic tail, and left diaphragm (Fig. 1a–e). Positron emission tomography (PET)–CT showed an accumulation of standardized uptake value (SUV) max of 4.1 and no obvious lymph node or distant metastasis (Fig. 2a, b). Based on the results of these imaging studies, malignant lymphoma (ML) or some kind of sarcoma was presented as the differential diagnosis. Colonoscopy showed an easily bleeding lesion with mucosal irregularities in the splenic flexure. A biopsy of the intestinal mucosa was performed at that time, and a histological examination suspected MS invading to the colon. Since the tumor was huge and had invaded the surrounding organs, especially the colon, and since it was most important to confirm the diagnosis, we decided to perform surgery. After informed consent by the patient, the surgical treatment followed; a total splenectomy, partial pancreatectomy, partial colectomy, left nephrectomy, left diaphragm partial resection, and cholecystectomy were performed (Fig. 3a–c). Postoperative histological examination revealed a diagnosis of MS in the primary of the spleen with presence of irregular karyotype cells, with immunohistochemical diffusely strong positivity for CD33 (Fig. 4), partially positivity for CD68, but negativity for CD3, CD20, CD34, CD56, myeloperoxidase (MPO), and naphthol AS-D chloroacetate esterase staining, and with a chromosomal test. The chromosome test showed an abnormal karyotype of 45, XY, del (1) (p13p22), add (6) (q13), add (10) (q22), -15, add (16) (q24)/ 46, XY, add (1) (q21), add (4) (q21), add (6) (q13), add (10) (q22), add (16) (q24), which indicated the complex karyotype, a poor prognostic type of AML. Bone marrow biopsy showed no abnormal cells, and ruled out leukocytosis. Postoperative complications included an ileus and intra-abdominal abscess on postoperative day 3, which was treated with drainage management and antibiotics, and finally, he was discharged home on postoperative day 62. After the surgery, he discussed the treatment strategy with hematologists, and it was decided that he would undergo bone marrow trephine biopsy. Immunophenotypic flow cytometry revealed no evidence of myeloid leukemia. He underwent umbilical cord blood transplantation, and he is now living without a sign of recurrence at 10 months after surgery.

## Discussion

MS is a rare disease, and primary nonleukemic MS of the spleen is particularly rare. A PubMed search of the period from 1963 to 2020 using the keywords “myeloid sarcoma” and “spleen” in English revealed only one reported case. Rao et al. reported a case of disseminated nonleukemic MS of the spleen involving the liver in a 5-month-old boy. Although he underwent surgery, he died of respiratory failure during the postoperative course. Here, we report for the first time a case of primary nonleukemic MS of the spleen that was successfully treated by surgery and hematopoietic stem cell transplantation.

Normally, MS is a complication in hematologic malignancies such as AML and myelodysplastic syndromes (MDS); it is reported that 78% of MS are complicated by AML and 20% by MDS, and less than 1% have no previous AML or MDS [5]. The misdiagnosis rate at initial diagnosis is reportedly high, with 47% of all MS cases being misdiagnosed as malignant lymphoma at the time of initial histopathological diagnosis [2]. Most often, MS is discovered after the diagnosis of malignant hematologic disease, and it is particularly rare for MS to be discovered as a result of mass formation prior to the development of a malignant hematologic disease: this occurs in about 1% of all MS cases [6].

Examinations for MS include the confirmation of cell type by bone marrow puncture, confirmation of markers including MPO, CD43, CD45, CD68, and other markers, and a search for distant metastases by PET–CT. Treatment for MS essentially requires anticancer therapy, according to leukemia [1, 3]. It has also been reported that both MS and developing AML were in remission when allogeneic hematopoietic stem cell transplantation and AML-like chemotherapy were administered within 4 months of diagnosis of MS [7]. Local treatment options for MS include radiation therapy, surgery, or both. Although surgery does not play an important role in patients with symptomatic MS, resection or debulking can be considered before starting chemotherapy [2, 3, 8]. In the present case, because of the huge tumor invading surrounding organs, and so as to confirm the diagnosis, we decided to perform surgery. Furthermore, as there was a high possibility of the patient’s disease progressing to AML in the future [1], the plan was to provide the treatment according to AML. Since bone marrow biopsy and PET–CT showed no evidence of leukocytosis, we concluded that the patient had achieved a complete response to surgical resection. Therefore, chemotherapy was not given as remission induction therapy, but allogenic hematopoietic stem cell transplantation was performed as consolidation therapy. There were no prodromal symptoms or findings at the time of transplantation, but considering the frequency of complications, it is recommended that allogeneic bone marrow transplantation be considered after the patient has reached first remission [9–14]. As an early treatment prior to the onset of hematological malignancies, a careful search for hematological malignancies with regular follow-up will be essential.

## Conclusions

We experienced a very rare case of primary spleen MS that was discovered without a hematologic malignancy. It is important to start treatment of MS as soon as possible, because early administration of treatment is expected to improve the prognosis of the disease.

## Data Availability

All data generated or analyzed during this study are included in this published article.

## Abbreviations

MS: Myeloid sarcoma
AML: Acute myeloid leukemia
CT: Computed tomography
MRI: Magnetic resonance imaging
PET–CT: Positron emission tomography–computed tomography
SUV: Standardized uptake value
ML: Malignant lymphoma
MPO: Myeloperoxidase
MDS: Myelodysplastic syndromes
